# Prevention, surveillance, and early detection of hepatocellular carcinoma – a Brazilian multidisciplinary consensus

**DOI:** 10.1590/0102-672020260000018e1947

**Published:** 2026-08-17

**Authors:** Felipe José Fernández COIMBRA, André Luís de GODOY, Paulo Henrique de Sousa FERNANDES, Heládio Feitosa e CASTRO, Rodrigo Nascimento PINHEIRO, Ilka de Fátima Santana Ferreira BOIN, Wellington ANDRAUS, Francisco TUSTUMI, Paulo Cezar Galvão do AMARAL, Carlos Eduardo BRANDÃO-MELLO, Alexcia Camila BRAUN, Maria Ignez BRAGHIROLI, Luis César BREDT, Carla CAPARROZ, Flair José CARRILHO, Guilherme Moura CUNHA, Alessandro Landskron DINIZ, Daniel Neves FORTE, José Huygens Parente GARCIA, Tércio GENZINI, Mauro Monteiro CORREIA, Cássio Virgílio Cavalcante de OLIVEIRA, Antonio Nocchi KALIL, Agnaldo Soares LIMA, Guilherme Lopes Pinheiro MARTINS, Cristina Melo ROCHA, Marcos Roberto de MENEZES, Joaquim Maurício da MOTTA-LEAL-FILHO, Munir MURAD, Renata D’Alpino PEIXOTO, Rafael Soares Nunes PINHEIRO, Marcelo Bruno de REZENDE, Héber Salvador de Castro RIBEIRO, Mario Rino MARTINS, Duilio Reis da ROCHA, Arthur Accioly ROSA, Luiz Arnaldo SZUTAN, Orlando Jorge Martins TORRES, Luiz Augusto Carneiro D’ALBUQUERQUE, Ângelo Alves de MATTOS, Anelisa Kruschewsky COUTINHO, Paulo HERMAN, Alexandre Ferreira OLIVEIRA

**Affiliations:** 1 A.C. Camargo Cancer Center, Department of Surgical Oncology, Abdominal Surgery – São Paulo (SP), Brazil.; 2 Universidade Federal de Uberlândia, Department of Surgical Oncology – Uberlândia (MG), Brazil.; 3 Instituto do Câncer do Ceará, Department of Surgical Oncology – Fortaleza (CE), Brazil.; 4 Hospital de Base do Distrito Federal, Department of Surgical Oncology – Brasília (DF), Brazil.; 5 Universidade Estadual de Campinas, Department of Surgery and Gastrocenter – Campinas (SP), Brazil.; 6 Universidade de São Paulo, Department of Gastroenterology – São Paulo (SP), Brazil.; 7 São Rafael Hospital/Hospital Rede D’Or, Department of Surgery of the Upper Digestive System – Salvador (BA), Brazil.; 8 Universidade Federal do Estado do Rio de Janeiro, Department of Internal Medicine – Rio de Janeiro (RJ), Brazil.; 9 Instituto do Câncer do Estado de São Paulo, Department of Oncology – São Paulo (SP), Brazil.; 10 Universidade Estadual do Oeste do Paraná, Department of Surgical Oncology and Hepatobilary Surgery – Cascavel (PR), Brazil.; 11 Hospital Beneficência Portuguesa de São Paulo, Department of Radiology – São Paulo (SP), Brazil.; 12 University of Washington, Department of Radiology – Seattle, USA.; 13 Universidade de São Paulo, Emergency Department – São Paulo (SP), Brazil.; 14 Universidade Federal do Ceará, Hospital Universitário Walter Cantídio, Department of Surgery, Liver Transplant Unit – Fortaleza (CE), Brazil.; 15 Hospital Leforte, Pancreas and Kidney Transplant Program – São Paulo (SP), Brazil.; 16 Instituto Nacional de Câncer, Department of Abdominopelvic Surgery – Rio de Janeiro (RJ), Brazil.; 17 Universidade Federal da Paraíba, Department of Surgery – João Pessoa (PB), Brazil.; 18 Irmandade Santa Casa de Misericórdia de Porto Alegre, Department of Hepato-Biliary-Pancreatic Surgery and Liver Transplantation – Porto Alegre (RS), Brazil.; 19 Universidade Federal de Minas Gerais, Department of Surgery – Belo Horizonte (MG), Brazil.; 20 Instituto do Câncer do Estado de São Paulo, Radiology Department – São Paulo (SP), Brazil.; 21 Universidade Federal do Amazonas, Department of Gastroenterology – Manaus (MN), Brazil.; 22 Universidade de São Paulo, Department of Radiology – São Paulo (SP), Brazil.; 23 Universidade Federal de Minas Gerais, Department of Oncology – Belo Horizonte (MG), Brazil.; 24 University of British Columbia, Department of Medical Oncology – Vancouver, Canada.; 25 Hospital Israelita Albert Einstein, Department of Health Sciences – São Paulo (SP), Brazil.; 26 Hospital de Câncer de Pernambuco, Department of Oncological Surgery – Recife (PE), Brazil.; 27 Universidade Federal do Ceará, Department of Oncology – Fortaleza (CE), Brazil.; 28 Hospital Santa Izabel and Oncoclínicas, Department of Radiation Oncology – Salvador (BA), Brazil.; 29 Santa Casa de São Paulo, Faculdade de Ciências Médicas, Department of Surgery – São Paulo (SP), Brazil.; 30 Universidade Federal do Maranhão, Department of Gastrointestinal Surgery – São Luis (MA), Brazil.; 31 Universidade Federal de Ciências da Saúde de Porto Alegre, Department of Gastroenterology and Hepatology – Porto Alegre (RS), Brazil.; 32 Rede Mater Dei, Department of Oncology – Salvador (BA), Brazil.; 33 Universidade Federal de Juiz de Fora, Department of Surgery – Juiz de Fora (MG), Brazil.

**Keywords:** Liver, Carcinoma, Hepatocellular, Consensus, Guidelines as Topic, Early Detection of Cancer, Epidemiology, Fígado, Carcinoma Hepatocelular, Consenso, Guias como Assunto, Detecção Precoce de Câncer, Epidemiologia

## Abstract

**Background:**

Hepatocellular carcinoma is a major cause of cancer-related mortality and represents a growing public health challenge.

**Aims:**

To develop multidisciplinary evidence-based recommendations for the prevention and early detection of hepatocellular carcinoma.

**Methods:**

This consensus was coordinated by the Brazilian Society of Surgical Oncology, with the participation of 13 additional national societies. A steering committee defined the key clinical questions addressing prevention, surveillance, and early detection strategies. Recommendation statements were informed by a comprehensive, non-systematic review of the literature conducted using PubMed, Scopus, and the Cochrane Library.

**Results:**

Eighteen recommendations reached consensus, covering three main domains: 1. Primary prevention, including HBV vaccination, timely antiviral therapy for viral hepatitis, and lifestyle interventions for metabolic dysfunction. 2. Secondary prevention, outlining risk-based surveillance strategies for patients with advanced fibrosis or cirrhosis due to hepatitis B virus, hepatitis C virus, or metabolic dysfunction–associated steatotic liver disease. 3. Operational standards for surveillance, emphasizing ultrasound as the cornerstone of monitoring, the importance of structured reporting, management of small nodules, and the selective use of cross-sectional imaging.

**Conclusions:**

These multidisciplinary recommendations provide practical, adaptable guidance for the prevention, surveillance, and early detection of hepatocellular carcinoma. This guidance document aims to reduce the burden of hepatocellular carcinoma and support better clinical outcomes across diverse healthcare settings.

## ARTICLE HIGHLIGHTS

This is the first article in a four-part series of the Brazilian multidisciplinary consensus on hepatocellular carcinoma.

This consensus provides evidence-based recommendations on prevention, surveillance, and early detection.

It integrates the available scientific literature with national clinical expertise to support and guide clinical decision-making.

## Summary

**CENTRAL MESSAGE:**

Prevention and early detection save lives in liver cancer. This Brazilian multidisciplinary consensus integrates the best scientific evidence and the expertise of specialists from across the country to more effectively guide the prevention, identification, and surveillance of hepatocellular carcinoma. By answering key clinical questions and offering practical, easy-to-use recommendations, this document helps physicians and healthcare teams provide better care to people at risk.

**PERSPECTIVES:**

This consensus provides practical guidance to improve the prevention and early detection of liver cancer in daily clinical care. The recommendations emphasize raising awareness of hepatocellular carcinoma, stimulating the development of broader surveillance programs, promoting earlier diagnosis, and improving outcomes for patients at risk for developing primary liver cancer.

## VISUAL ABSTRACT

**Figure:**
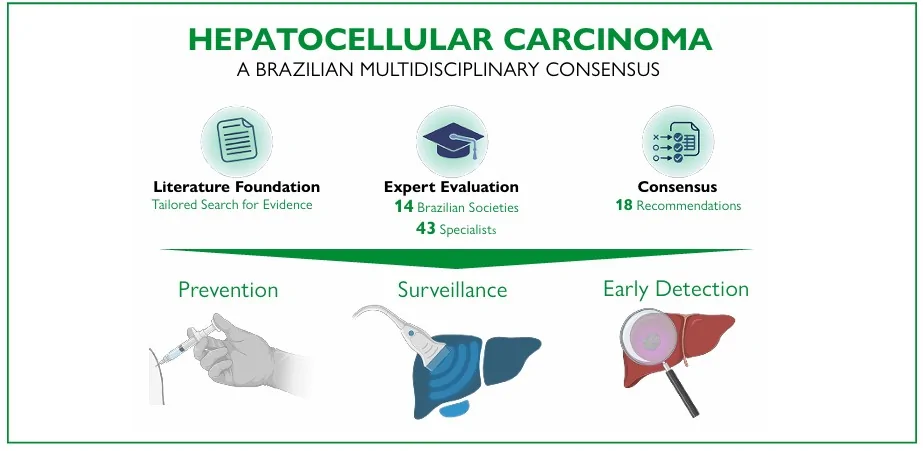

## RESUMO

**Racional:**

O carcinoma hepatocelular (CHC) é uma das principais causas de mortalidade relacionada ao câncer e representa um crescente desafio para a saúde pública.

**Objetivos:**

Desenvolver recomendações multidisciplinares baseadas em evidências para a prevenção e detecção precoce do CHC.

**Métodos:**

Este consenso foi coordenado pela Sociedade Brasileira de Oncologia Cirúrgica, com a participação de outras 13 sociedades nacionais. Um comitê diretivo definiu as principais questões clínicas abordando estratégias de prevenção, vigilância e detecção precoce. As recomendações foram elaboradas com base em uma revisão abrangente e não sistemática da literatura, realizada utilizando PubMed, Scopus e a Biblioteca Cochrane.

**Resultados:**

Dezoito recomendações foram obtidas por consenso, abrangendo três domínios principais: (1) Prevenção primária, incluindo vacinação contra o VHB, terapia antiviral oportuna para hepatite viral e intervenções no estilo de vida para disfunção metabólica. (2) Prevenção secundária, delineando estratégias de vigilância baseadas em risco para pacientes com fibrose avançada ou cirrose devido a VHB, VHC ou MASLD. (3) Padrões operacionais para vigilância, enfatizando a ultrassonografia como a base do monitoramento, a importância da elaboração de relatórios estruturados, o manejo de pequenos nódulos e o uso seletivo de exames de imagem seccionais.

**Conclusões:**

Estas recomendações multidisciplinares fornecem orientações práticas e adaptáveis para a prevenção, vigilância e detecção precoce do CHC. Este documento de orientação visa reduzir a carga do CHC e apoiar melhores resultados clínicos em diversos contextos de saúde.

## INTRODUCTION

 Liver cancer represents a major global health challenge. According to GLOBOCAN 2020, it is the seventh most common malignancy and the second leading cause of cancerrelated death worldwide^124^. Hepatocellular carcinoma (HCC) constitutes 75–85% of primary liver tumors^87^. 

 In Brazil, although epidemiological information remains limited, data from the National Cancer Institute of Brazil (INCA) indicate that liver cancer ranks 15th in incidence, with the highest rates observed in the South region for both sexes^95^. Between 2001 and 2015, more than 120,000 deaths were recorded due to malignant liver neoplasms, predominantly in men (56.9%)^29^. Mortality varies regionally, with the North presenting the highest rates overall, while female mortality peaks in the Northeast and male mortality in the South^29^. 

 In Brazil, viral hepatitis remains the leading cause of HCC. Nearly 50% of cases are linked to the hepatitis B virus (HBV) and around 25% to the hepatitis C virus (HCV)^41,44^. HCV-infected individuals have a 17-fold higher risk of developing HCC^48^, and coinfection with HBV accelerates progression to cirrhosis and hepatic decompensation^19^. Infection by the hepatitis D virus, which relies on HBV for replication, further increases the risk of HCC when compared with HBV alone^57,101^. 

 Metabolic dysfunction–associated risk factors are becoming increasingly relevant^45^. Diabetes, overweight, and obesity have an association with the development of metabolic dysfunction-associated steatotic liver disease (MASLD) and a two-fold increase in HCC risk, even in the absence of cirrhosis^33,92^. With the rapid rise of obesity and diabetes, MASLD is projected to become a leading cause of HCC in the future^7^. Excessive alcohol consumption (>40–60 g/day) is also a major contributor^28^, displaying a linear dose-response relationship and doubling HCC risk in individuals with HCV coinfection^42^. Environmental exposures, especially aflatoxin contamination of poorly stored grains and nuts, remain a dominant etiologic factor in parts of Asia and Africa, due to carcinogenic mutations such as those affecting TP53^11,28^. Additionally, rare hereditary metabolic and cholestatic disorders are associated with an increased risk^58^. 

 Given the diversity of etiologic profiles, regional disparities in incidence and mortality, and varying levels of access to surveillance and treatment, context-specific guidance is crucial. In this setting, a Brazilian multidisciplinary consensus is essential to translate high-quality scientific evidence into standardized recommendations. The objective of this consensus is to synthesize the most current national and international literature on the prevention, surveillance, and early detection of HCC, to provide structured, multidisciplinary guidance, and to serve as the first article in a four-part series developed by this Brazilian multidisciplinary consensus addressing the full continuum of HCC care. 

## METHODS

### Study design

 This consensus was developed under the leadership of the Brazilian Society of Surgical Oncology, in collaboration with 13 additional Brazilian medical societies, thereby ensuring comprehensive, multidisciplinary representation (Table 1). The initiative focused on developing evidence-based recommendations to address the prevention, surveillance, and early detection of HCC. 

**Table 1:** List of the medical societies that participated in the consensus.

Participating Medical Societies
Brazilian Society of Oncological Surgery^*^
Brazilian Society of Clinical Oncology
Brazilian College of Surgeons
Brazilian College of Hepato-Pancreato-Biliary Surgery
Brazilian College of Digestive Surgery
Brazilian Society of Interventional Radiology and Endovascular Surgery
Brazilian Society of Radiotherapy
Brazilian Association of Organ Transplantation
Brazilian College of Radiology and Diagnostic Imaging
Brazilian Society of Hepatology
Brazilian Society of Pathology
Brazilian Federation of Gastroenterology
National Academy of Palliative Care

* Host organization.

### Expert panel

**Figure 1:**
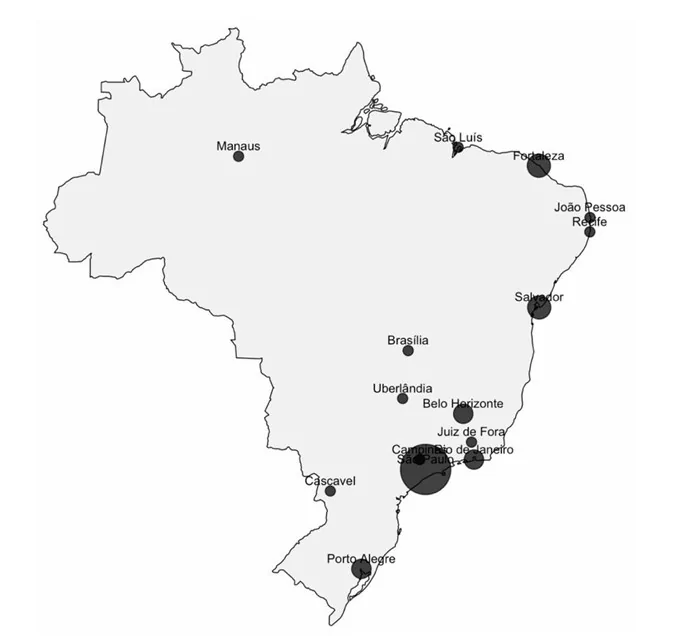
Geographic distribution of Brazilian cities participating in the Hepatocellular carcinoma consensus. Circle size is proportional to the frequency of participating experts in each city.

 The expert panel consisted of 43 specialists appointed by the participating societies (Figure 1). Panel members were selected based on their expertise in hepatology, oncology, hepatopancreatobiliary surgery, liver transplantation, radiology, gastroenterology, and related fields. The panel was responsible for evaluating scientific evidence, supporting the development of rationales, and issuing recommendations based on the strength of the evidence. 

### Formulation of clinical questions

 Initially, a scientific steering committee identified key topics and designed clinically relevant questions about HCC surveillance strategies, surveillance criteria, and preventive interventions. These questions were organized to reflect clinical decisionmaking needs across different settings and were refined through preliminary meetings with representatives of all participating societies to guarantee a broad, cross-disciplinary perspective. 

### Literature review

 A targeted narrative review of the literature was conducted to inform the development of the clinical questions and recommendations. Searches were performed in PubMed, Scopus, and the Cochrane Library, focusing on studies published over the past 30 years. The search aimed to identify clinically relevant evidence. 

 Eligible sources included clinical studies in humans, such as observational studies, randomized and non-randomized trials, systematic reviews, practice guidelines, and prior consensus statements relevant to HCC prevention, surveillance, and early detection. Preclinical, experimental, and animal studies were not prioritized unless considered essential for contextual understanding. 

 Publications were selected based on their relevance to the predefined topics and clinical applicability. The steering committee reviewed and synthesized the evidence qualitatively, ensuring consistency of interpretation and alignment between the supporting data and the final recommendations. 

### Consensus process and voting rounds

 Following expert deliberations, the synthesized clinical questions and draft recommendations were circulated to representatives of the 14 participating societies through an electronic voting platform as part of an adapted Delphi process^94^. Participants independently rated each item and provided written feedback. After the first round, aggregated results and anonymized comments were discussed in structured online meetings, and statements were revised to enhance clarity, scientific accuracy, and clinical applicability. 

 A second round of electronic voting was then conducted using the revised items. Consensus was defined a priori as an approval rate of ≥80%, based on the response options “completely accept” or “accept with reservations.” Statements reaching this threshold in the second round were considered to have achieved consensus and were incorporated into the final document, whereas those that did not meet the predefined criterion were excluded from the final recommendations.. 

### Evidence level and recommendation grading

 The quality of evidence and the strength of recommendations were assessed using the UpToDate^®^ grading system^136^. Evidence quality reflects confidence in effect estimates and is categorized as high, moderate, or low. Evidence quality reflects confidence in estimates and is categorized as high, moderate, or low, ranging from consistent results derived from well-conducted randomized controlled trials, systematic reviews, evidence based primarily on observational studies or trials with significant methodological limitations. Recommendations are classified as strong or weak according to the balance between benefits and risks, the quality of supporting evidence, and the degree of certainty surrounding outcomes. Strong recommendations indicate that benefits clearly outweigh risks and apply to most patients in most clinical scenarios. In contrast, weak recommendations reflect uncertainty, suggesting that alternative approaches may be equally reasonable (Table 2) 

**Table 2:** UpToDate^®^ grading system for quality of evidence and grade of recommendation.

Grade of recommendation	Quality of supporting evidence	Clarity of risk/benefit	Implications
Strong	High	Benefits clearly outweigh risks and burdens, or vice versa.	A strong recommendation applies to most patients in most circumstances without reservation. Clinicians should follow a strong recommendation unless a clear and compelling rationale for an alternative approach is present.
	Moderate	Benefits clearly outweigh risks and burdens, or vice versa.	A strong recommendation applies to most patients. Clinicians should follow a strong recommendation unless a clear and compelling rationale for an alternative approach is present.
	Low	Benefits appear to outweigh risks and burdens, or vice versa.	A strong recommendation applies to most patients. Some of the evidence base supporting the recommendation is, however, of low quality.
Weak	High	Benefits closely balanced with risks and burdens.	Weak recommendation, best action may differ depending on the circumstances, the patients, or their societal values.
	Moderate	Benefits closely balanced with risks and burdens. Some uncertainty in the estimates of benefits, risks, and burdens.	Weak recommendation, alternative approaches likely to be better for some patients under some circumstances.
	Low	Uncertainty in the estimates of benefits, risks, and burdens. Ben efits may be closely balanced with risks and burdens.	Very weak recommendation; other alternatives may be equally reasonable.

## RESULTS

### Expert characteristics

 A total of 43 Brazilian experts participated in this national multidisciplinary consensus on HCC. The expert panel developed 18 evidence-based recommendations addressing key aspects of prevention, surveillance, and early diagnosis of HCC. The final recommendations are presented in Table 3. 

**Table 3:** List of recommendations alongside the level of evidence and recommendation grades.

Recommendation		Evidence level	Recommendation grade
1	Universal HBV vaccination — preferably initiated at birth — should be implemented as a primary prevention strategy to reduce the incidence of HBV-related HCC.	High	Strong
2	Antiviral treatment with nucleotide analogs should be initiated in patients with chronic HBV who have high serum HBV DNA levels (>10^4^ copies/mL or >2,000 IU/mL), elevated alanine aminotransferase, HBeAg positivity, and/or cirrhosis, as long-term viral suppression reduces the risk of HCC and disease progression. There is no recommendation for routine antiviral treatment in patients in the immune-tolerant phase solely to prevent HCC.	Moderate	Weak
3	Early treatment of chronic HCV infection with DAA is recommended to prevent the progres sion to advanced fibrosis or cirrhosis and reduce the risk of HCC. The benefit is most evident in patients who achieve sustained virologic response.	High	Strong
4	DAA therapy improves the quality of life and helps prevent hepatic decompensation in patients with cirrhosis. However, the impact of DAAs on reducing the risk of HCC recur rence remains incompletely elucidated, as does the appropriate timing for initiating antiviral treatment. Antiviral therapy may be offered to patients with HCC who have previously been treated with curative intent, given the uncertain impact on recurrence risk, while recogniz ing the clear benefits of viral eradication in maintaining liver function. While current evidence does not conclusively demonstrate a reduction in recurrence risk, delaying antiviral therapy solely due to concerns about recurrence is not supported.	Moderate	Weak
5	Patients with HCV-related HCC treated with curative intent should undergo indefinite surveillance with dynamic contrast-enhanced imaging (either multiphasic abdominal MRI or triphasic CT) every 3 to 6 months, given the persistent risk of recurrence related to underly ing liver disease and the field effect of hepatocarcinogenesis.	Moderate	Weak
6	Postoperative antiviral therapy is recommended for all patients with HBV-related HCC un dergoing curative-intent treatment, including liver resection or transplantation, regardless of baseline viral load.	High	Strong
7	Although no randomized controlled trials have confirmed a direct benefit of glycemic control in preventing HCC, diabetes management is strongly recommended in patients with fatty liver disease, especially when additional risk factors for cirrhosis are present.	Low	Strong
8	For overweight or obese patients with nonalcoholic fatty liver disease, lifestyle interventions, such as dietary changes and regular physical activity, are recommended to promote weight loss, improve metabolic parameters, and potentially reduce the risk of HCC.	High	Strong
9	To reduce the risk of HCC, it is recommended to avoid excessive alcohol consumption. Ex cessive alcohol use is associated with hepatic steatosis, fibrosis progression, and HCC.	High	Strong
10	Coffee consumption should be encouraged in patients with chronic liver disease as an ac cessible and potentially protective strategy against HCC. Metformin use is recommended in diabetic patients with chronic liver disease for its glycemic benefits and potential reduction in HCC risk. Despite promising observational data, statins should not be routinely used solely for HCC prevention until higher-quality evidence confirms their benefit in this setting.	Moderate	Weak
11	HCC surveillance is recommended for all patients with advanced fibrosis (F3) or cirrhosis (F4), regardless of etiology. In chronic HBV, surveillance is also indicated in F0–F2 patients with high-risk features (e.g., older age, male sex, high HBV DNA, family history, or Asian/Af rican ancestry). In selected F0–F2 cases with overlapping risks, individualized surveillance may be considered.	High	Strong
12	HCC surveillance is recommended for patients with MASLD who have biopsy-proven or non-invasively diagnosed advanced fibrosis (F3) or cirrhosis (F4).	High	Strong
13	Abdominal ultrasound is the first-line imaging modality for HCC surveillance.	Moderate	Strong
14	Serum alpha-fetoprotein should not be used as a standalone method for HCC surveillance. However, its combination with abdominal ultrasound may increase sensitivity for early tumor detection and can be considered, particularly in high-risk patients.	Moderate	Weak
15	HCC surveillance should be performed every 6 months.	Moderate	Strong
16	Ultrasound reports used for HCC surveillance in high-risk patients should be structured and standardized according to LI-RADS US®, explicitly documenting any acoustic limitations that may impair liver visualization and lesion detection. The report must clearly state the presence or absence of focal hepatic lesions and describe nodules with indeterminate morphology, specifying whether they are smaller or larger than 1 cm. In high-risk patients, ultrasound alone should not be used to establish a definitive diagnosis of hepatic hemangioma, and ad ditional imaging evaluation is required. Color and spectral Doppler assessment of hepatic and portal vascular structures should be routinely included to evaluate portal hypertension and to identify possible tumor-related or hematologic thrombosis.	Low	Strong
17	In patients with nodules smaller than 1 cm detected on ultrasound, follow-up should be performed at 3–4-month intervals for the first 2 years. If the nodule size remains stable after this period, follow-up can be resumed every 6 months.	Low	Strong
18	In patients with cirrhosis, MRI or CT may be considered as a surveillance modality for HCC when ultrasound has technical limitations that compromise its diagnostic performance.	Low	Strong

HCC: Hepatocellular carcinoma; HBV: Hepatitis B virus; HBeAg: Hepatitis B e antigen; HCV: Hepatitis C virus; DAA: Direct-acting antiviral; MASLD: Metabolic dysfunction-associated steatotic liver disease; MRI: Magnetic resonance imaging; CT: Computed tomography.

### What is the importance of universal hepatitis B vaccination in preventing hepatocellular carcinoma?

 Chronic HBV infection is a well-established risk factor for HCC, accounting for a substantial proportion of global cases, particularly in endemic regions^16^. Although antiviral therapy can reduce viral replication and lower HCC risk, HBV eradication remains difficult, with functional cure achieved in a minority of patients^76,77^. Most individuals require long-term or lifelong treatment. 

 In contrast, universal HBV vaccination, particularly when administered at birth, prevents chronic infection and is a highly effective primary prevention measure. Longitudinal data from Taiwan demonstrate a marked decline in childhood HCC incidence within a decade of implementing a national vaccination program^16,64^, findings that have been consistently replicated in other populations^20,52,60,88,102,140^. These results highlight the long-term population-level impact of vaccination on reducing HCC burden. 

### Recommendation 1

 Universal HBV vaccination — preferably initiated at birth — should be implemented as a primary prevention strategy to reduce the incidence of HBV-related HCC. Evidence level: High. Recommendation grade: Strong. 

### What is the importance and impact of early treatment of hepatitis B virus infection on preventing Hepatocellular carcinoma?

 In patients with chronic HBV, several factors are consistently associated with an increased risk of HCC and disease progression, including high serum HBV DNA levels (>10^4^ copies/mL or >2,000 IU/mL), elevated alanine aminotransferase (ALT), hepatitis B e antigen (HBeAg) positivity, and the presence of cirrhosis^53^. In this context, long-term suppression of viral replication with nucleotide analogs has been shown to significantly reduce the incidence of HCC and overall mortality in patients with immune-active disease or cirrhosis^21,79^. 

 The role of antiviral therapy in early disease stages, particularly in immune-tolerant patients with normal ALT levels and no significant fibrosis, remains controversial. Retrospective cohort studies from Asia have suggested higher long-term risks of HCC and liver-related mortality among untreated immune-tolerant individuals^69,118^. In contrast, prospective data from the Hepatitis B Research Network cohort in North America demonstrated a very low incidence of adverse clinical outcomes among carefully selected immune-tolerant patients under close surveillance^80^. These divergent findings highlight ongoing uncertainty regarding the benefit of antiviral treatment in this population for HCC prevention. 

### Recommendation 2

 Antiviral treatment with nucleotide analogs should be initiated in patients with chronic HBV who have high serum HBV DNA levels (>10^4^ copies/mL or >2,000 IU/mL), elevated alanine aminotransferase, HBeAg positivity, and/or cirrhosis, as long-term viral suppression reduces the risk of HCC and disease progression. There is no recommendation for routine antiviral treatment in patients in the immune-tolerant phase solely to prevent HCC.Evidence level: Moderate. Recommendation grade: Weak. 

### What is the importance and impact of early treatment of hepatitis C virus infection on preventing hepatocellular carcinoma?

 Chronic HCV infection is a well-established cause of HCC, and the risk increases with the progression of liver fibrosis^132^. The advent of direct-acting antivirals (DAAs) has significantly transformed the management of HCV, offering sustained virologic response (SVR) rates of 95–100% with favorable safety profiles. 

 Achieving SVR with DAA therapy is associated with a substantial reduction in the incidence of HCC, particularly when treatment occurs before the development of advanced fibrosis or cirrhosis^55,62^. 

 While early retrospective studies raised concerns about a possible association between DAAs and increased HCC risk or recurrence, subsequent large-scale analyses and prospective cohorts refuted this hypothesis and demonstrated the protective role of antiviral therapy^63,134^. A pivotal French cohort study^14^ confirmed that patients treated with DAAs had reduced risks of all-cause mortality and HCC, including those with cirrhosis. Notably, this benefit was seen only among patients who achieved SVR, while those without virological cure maintained a high risk of liver related outcomes. 

### Recommendation 3

 Early treatment of chronic HCV infection with DAA is recommended to prevent the progression to advanced fibrosis or cirrhosis and reduce the risk of HCC. The benefit is most evident in patients who achieve sustained virologic response. Evidence level: High. Recommendation grade: Strong. 

### What is the impact of hepatitis C virus infection treatment on preventing recurrence of hepatocellular carcinoma treated with curative intent (tertiary prevention related to hepatitis C virus)?

 A prospective cohort study by Cabibbo et al. showed that DAAs significantly improved overall survival and reduced the risk of hepatic decompensation in 163 patients with compensated HCV-related cirrhosis and successfully treated HCC with curative resection or ablation. However, the risk of HCC recurrence was not significantly reduced (HR 0.7; 95%CI 0.44–1.13; p=0.15)^13,57^. 

### Recommendation 4

 DAA therapy improves the quality of life and helps prevent hepatic decompensation in patients with cirrhosis. However, the impact of DAAs on reducing the risk of HCC recurrence remains incompletely elucidated, as does the appropriate timing for initiating antiviral treatment. Antiviral therapy may be offered to patients with HCC who have previously been treated with curative intent, given the uncertain impact on recurrence risk, while recognizing the clear benefits of viral eradication in maintaining liver function. While current evidence does not conclusively demonstrate a reduction in recurrence risk, delaying antiviral therapy solely due to concerns about recurrence is not supported. Evidence level: Moderate. Recommendation grade: Weak. 

### What is the recommended posttreatment surveillance strategy for patients with hepatitis C virus related hepatocellular carcinoma treated with curative intent?

 Even after achieving an SVR through DAA therapy, patients with HCV-related cirrhosis remain at risk for HCC recurrence due to the persistent effects of chronic liver injury and the concept of a “field cancerization” effect in cirrhotic livers^55,132^. Studies have shown that the risk of recurrence after curative-intent treatment, such as resection or ablation, remains high, particularly in the first two years post-treatment^13,103^. 

 The persistence of recurrence risk underscores the need for long-term surveillance to enable early detection of potentially treatable lesions. However, the optimal surveillance intervals remain a subject of ongoing debate. 

### Recommendation 5

 Patients with HCV-related HCC treated with curative intent should undergo indefinite surveillance with dynamic contrast-enhanced imaging (either multiphasic abdominal MRI or triphasic CT) every 3 to 6 months, given the persistent risk of recurrence related to underlying liver disease and the field effect of hepatocarcinogenesis.Evidence level: Moderate. Recommendation grade: Weak. 

### What is the impact of hepatitis B virus infection treatment on preventing hepatocellular carcinoma recurrence (tertiary prevention related to hepatitis B virus)?

 In patients with HBV-related HCC, high HBV DNA levels and greater hepatic inflammatory activity are among the strongest predictors of postoperative recurrence^18,119^. Several retrospective and prospective studies support the role of antiviral therapy with nucleos(t)ide analogs in reducing the risk of recurrence and improving survival following curative-intent treatments. 

 Early retrospective studies demonstrated that patients with high HBV DNA levels benefited from antiviral therapy after curative liver resection^22,138^. Subsequent randomized clinical trials confirmed these findings. In one trial, Yin et al.^141^ showed that postoperative antiviral therapy improved recurrence-free and overall survival. Similarly, Huang et al.^50^ found that adefovir reduced late recurrence in patients with HBV DNA >2,000 IU/mL after resection. More recently, telbivudine-based therapy was shown to reduce recurrence and improve 5-year overall survival (64 vs. 44%)^51^. 

 In the liver transplant setting, HBV recurrence is more common among recipients with HCC than those without (2–35 vs. 1–9.7%) and has been associated with increased risk of tumor recurrence. Therefore, antiviral prophylaxis is critical to minimize both viral and oncologic relapse^30,130^. 

### Recommendation 6

 Postoperative antiviral therapy is recommended for all patients with HBV-related HCC undergoing curative-intent treatment, including liver resection or transplantation, regardless of baseline viral load.Evidence level: High. Recommendation grade: Strong. 

### What is the importance of diabetes control in patients with fatty liver disease for the prevention of hepatocellular carcinoma?

 The association between diabetes mellitus and HCC has been consistently demonstrated across multiple epidemiological studies^5,17,32,46,54,58,71,74,110,1^. Yang et al.^139^, analyzing 14 prospective cohort studies, found that diabetes increased the risk of HCC by 90% (RR 1.9; 95%CI 1.2–2.3). Wang et al.^133^ reported a pooled relative risk of 2.2 (95%CI 1.7–3.0) for HCC among diabetic patients in a systematic review. 

 Despite this strong epidemiological association, no randomized controlled trials have evaluated whether intensive glycemic control directly reduces the risk of HCC. Nonetheless, given the significant overlap between diabetes, metabolic syndrome, fatty liver disease, and progression to cirrhosis, glycemic control remains a fundamental component of comprehensive risk reduction strategies in patients with fatty liver disease. 

### Recommendation 7

 Although no randomized controlled trials have confirmed a direct benefit of glycemic control in preventing HCC, diabetes management is strongly recommended in patients with fatty liver disease, especially when additional risk factors for cirrhosis are present. Evidence level: Low. Recommendation grade: Strong. 

### What is the role of lifestyle interventions and weight loss in reducing hepatocellular carcinoma risk in patients with nonalcoholic fatty liver disease?

 MASLD is increasingly recognized as a relevant risk factor for HCC, including in non-cirrhotic individuals^7,91^. The mechanisms of hepatocarcinogenesis in MASLD are multifactorial and not yet fully understood, but metabolic dysfunction — particularly in the presence of obesity, type 2 diabetes, dyslipidemia, and hypertension — plays a central role^72,100,122^. 

 Although the direct impact of weight loss on HCC incidence remains to be definitively established, multiple studies have demonstrated that lifestyle interventions, especially those leading to meaningful weight reduction, improve hepatic steatosis and can promote histological resolution of MASLD and regression of fibrosis^73,100,131^. In the Cuban cohort by Vilar-Gomez et al.^131^, ≥10% weight loss led to MASLD resolution in 90% of patients and fibrosis regression in 45%. Additionally, the Singapore Chinese Health Study associated healthy lifestyle habits — such as physical activity, Mediterranean diet, nonsmoking, low alcohol intake, and adequate sleep — with a reduced risk of HCC^82^. These findings support a preventive role for structured lifestyle modifications, particularly in patients with MASLD and metabolic risk factors. 

### Recommendation 8

 For overweight or obese patients with nonalcoholic fatty liver disease, lifestyle interventions, such as dietary changes and regular physical activity, are recommended to promote weight loss, improve metabolic parameters, and potentially reduce the risk of HCC. Evidence level: High. Recommendation grade: Strong. 

### How important is reducing alcohol consumption in preventing hepatocellular carcinoma?

 Excessive alcohol intake is a well-established risk factor for liver disease progression, particularly in individuals with underlying metabolic dysfunction or viral hepatitis. In patients with MASLD, excessive alcohol consumption has been independently associated with increased hepatic steatosis, liver inflammation, and accelerated fibrosis progression^31,72,97^. In a Swedish cohort, excessive alcohol use (defined as >60 g/day for men and >48 g/day for women) was linked to significantly higher rates of fibrosis progression in MASLD patients (47 vs. 11%)^31^. 

 Evidence also suggests that even light to moderate alcohol consumption may negatively impact liver health in at-risk populations. Ajmera et al.^2^ and VanWagner et al.^129^ showed that modest alcohol intake worsened steatosis, increased liver enzyme levels, and reduced the likelihood of MASLD resolution. Additionally, synergistic effects have been documented between alcohol and other HCC risk factors, such as viral hepatitis, diabetes, and obesity^28,47,81^. 

 Although prospective studies evaluating HCC incidence in light/moderate drinkers are lacking, the available evidence supports alcohol reduction — especially in individuals with underlying liver disease — as a key preventive strategy. 

### Recommendation 9

 To reduce the risk of HCC, it is recommended to avoid excessive alcohol consumption. Excessive alcohol use is associated with hepatic steatosis, fibrosis progression, and HCC. Evidence level: High. Recommendation grade: Strong. 

### What is the role of statins, metformin, and coffee consumption in hepatocellular carcinoma prevention?

 Observational studies have suggested a protective association between the use of statins and the risk of HCC in patients with chronic liver disease, particularly those with viral hepatitis. Five recent meta-analyses^37,56,59,75,143^ consistently reported a reduction in HCC incidence among statin users. In the largest analysis, Facciorusso et al.^37^ evaluated more than 1.9 million individuals and found that lipophilic statins were associated with a 51% reduction in HCC risk (HR 0.49; 95%CI 0.39–0.62). Subgroup analyses confirmed this benefit across patients with or without HBV/HCV infection, diabetes, cirrhosis, and regardless of age or sex. However, all supporting studies are observational, and no randomized controlled trials have yet confirmed a causal relationship. Therefore, the literature still lacks high-quality studies that adequately weigh the benefits against potential adverse effects and does not yet provide strong evidence to support the use of statins solely for chemoprevention. 

 Metformin has also been associated with a reduced risk of HCC in diabetic patients with chronic liver disease. Several meta-analyses, including Zhou et al.^144^, have shown that metformin use significantly reduces HCC risk compared with insulin or sulfonylureas (RR 0.49; 95%CI 0.25–0.97). A meta-analysis by Ma et al.^83^, involving over 550,000 individuals with diabetes, also showed improved survival among HCC patients treated with metformin (OR 0.52; 95%CI 0.40–0.68; p<0.001). Given these findings and the overall benefit in glycemic control, metformin should be prioritized in antidiabetic regimens for these patients. 

 Coffee consumption has been consistently associated with a dose-dependent reduction in HCC risk, regardless of underlying liver disease etiology. Kennedy et al.^67^ analyzed 18 cohort studies (over 2 million participants and 2,905 cases) and found that each additional two cups of coffee per day reduced HCC risk by 35% (RR 0.65; 95%CI 0.59–0.72). Similar findings were observed across subgroups with chronic liver disease, alcohol use, obesity, and diabetes. While data on decaffeinated coffee are inconclusive^8,67^, evidence supports recommending regular coffee consumption as a low-cost, accessible, and potentially protective measure in individuals with chronic liver disease^15,35^. 

### Recommendation 10

 Coffee consumption should be encouraged in patients with chronic liver disease as an accessible and potentially protective strategy against HCC. Metformin use is recommended in diabetic patients with chronic liver disease for its glycemic benefits and potential reduction in HCC risk. Despite promising observational data, statins should not be routinely used solely for HCC prevention until higher-quality evidence confirms their benefit in this setting. Evidence level: Moderate. Recommendation grade: Weak. 

### Which patients with viral hepatitis should undergo hepatocellular carcinoma surveillance?

 Chronic viral hepatitis infection carries a high likelihood of progression to chronic hepatitis, cirrhosis, and ultimately HCC in a substantial proportion of cases. Longitudinal cohort data suggest that 20–25% of chronic hepatitis patients develop cirrhosis over 20 to 30 years, with an annual risk of HCC ranging from 1 to 2% in cirrhotic patients^99^. In chronic HBV infection acquired in adulthood, approximately 5–10% of patients progress to cirrhosis, with a subsequent risk of developing HCC^84^. 

 In theory, counseling for surveillance and monitoring of patients with chronic viral hepatitis and potential risk for HCC should be prioritized for those with more advanced stages of liver fibrosis (F3–F4) and/or cirrhosis. However, a minority of patients with chronic viral hepatitis — particularly those with HBV infection and/or additional risk factors — can develop HCC even in the presence of only mild or moderate fibrosis. These risk factors include diabetes, metabolic syndrome, HIV or other viral coinfections, family history of HCC, iron overload, and older age. In such cases, surveillance strategies may be individualized, especially when multiple risk factors overlap. In HBV specifically, certain populations — including males over 40, individuals of Asian or African descent, patients with high HBV DNA levels, and those with a family history of HCC — are considered at elevated risk and may benefit from HCC surveillance regardless of fibrosis stage^39^. 

### Recommendation 11

 HCC surveillance is recommended for all patients with advanced fibrosis (F3) or cirrhosis (F4), regardless of etiology. In chronic HBV, surveillance is also indicated in F0–F2 patients with high-risk features (e.g., older age, male sex, high HBV DNA, family history, or Asian/African ancestry). In selected F0–F2 cases with overlapping risks, individualized surveillance may be considered. Evidence level: High. Recommendation grade: Strong. 

### Should patients with metabolic dysfunction-associated steatotic liver disease and significant fibrosis undergo hepatocellular carcinoma surveillance?

 Brazilian data support the close relationship between metabolic dysfunction and HCC. In a cohort of 110 patients with HCC attributed to MASLD, obesity was present in 52.7%, diabetes in 73.6%, dyslipidemia in 41%, and metabolic syndrome in 57.2%^25^. Among those with histological confirmation, 61.5% had MASLD-related cirrhosis and 27% showed fibrosis stage 1–3, while a minority exhibited HCC in the absence of fibrosis. In a large U.S. cohort of patients with MASLD, the incidence of HCC reached 1.06 per 100 person-years^114^. Importantly, hepatic fibrosis is the key prognostic determinant, with advanced fibrosis independently associated with liver-related events and overall mortality. In an Asian cohort of more than 6,500 patients with ultrasound-defined MASLD, an APRI >1.5 significantly predicted HCC risk over a median follow-up of 5.6 years^66^. 

 Although only 10–30% of MASLD cases progress to cirrhosis, HCC can occasionally develop in the absence of advanced fibrosis^49^. Nonetheless, the incidence of HCC in non-cirrhotic MASLD remains extremely low — approximately 0.008 per 100 person-years^3,63^ — which does not justify routine surveillance in this subgroup. In contrast, individuals with advanced fibrosis (F3) or cirrhosis (F4), whether diagnosed through biopsy or non-invasive tools, carry a substantially higher risk of HCC and are appropriate candidates for surveillance. 

 Various alternative methods have been proposed to stage MASLD, including biomarkers (e.g., CK-18 fragments), genetic polymorphisms (e.g., PNPLA3 I148M), and metabolomic/lipidomic-based models, alongside advanced imaging techniques^34^. To reduce diagnostic uncertainty, current recommendations support combining two complementary fibrosis assessments — a serologic and an imaging-based. When both suggest advanced fibrosis or cirrhosis, HCC surveillance is considered justified. Conversely, in the absence of advanced fibrosis, surveillance is not routinely indicated due to the low incidence of HCC^63^. 

### Recommendation 12

 HCC surveillance is recommended for patients with MASLD who have biopsy-proven or non-invasively diagnosed advanced fibrosis (F3) or cirrhosis (F4). Evidence level: High. Recommendation grade: Strong. 

### What is the most appropriate imaging modality for hepatocellular carcinoma surveillance?

 Over the years, several cohort studies^10,23,24,111,137^ and costeffectiveness analyses^6,78,113^ have assessed the benefit of ultrasound-based HCC surveillance and helped define the target populations. A meta-analysis published in 2009 reported that abdominal ultrasound had a sensitivity ranging from 60–80% and a specificity of over 90% for early-stage HCC detection^116^. More recently, a 2018 meta-analysis including 32 studies from 1990 to 2016 estimated a pooled sensitivity of 84% (95%CI 76–92%) for HCC, but only 47% (95%CI 33–61%) for earlystage tumors. Among the nine studies that directly evaluated early-stage detection, sensitivity was 53% (95%CI 35–70%) and specificity was 91% (95%CI 86–94%)^128^. The reduced sensitivity of ultrasound surveillance for HCC detection is closely associated with underlying liver conditions that impair acoustic penetration and lesion conspicuity, particularly hepatic steatosis related to MASLD and alcohol-related liver disease. In these settings, increased parenchymal heterogeneity substantially limits image quality and diagnostic performance, contributing to early-stage tumors being missed^40^. 

 One of the main disadvantages of ultrasound is its marked operator dependence, which can lead to substantial variability in examination quality and diagnostic performance. To improve reporting standardization and communication between imaging specialists and referring clinicians, the ultrasound Liver Imaging Reporting and Data System (US LI-RADS) was introduced. In addition to categorizing focal liver observations, this framework has a visualization score that reflects the overall adequacy of the examination for surveillance purposes. Patients are classified as having no or minimal limitations (score A), moderate limitations (score B), or severe limitations (score C), the latter indicating that ultrasound surveillance may be unreliable. A large meta-analysis including more than 25,000 examinations reported that moderate or severe visualization limitations occur in a substantial proportion of studies, particularly among patients with cirrhosis related to metabolic liver disease and obesity^61^. Importantly, these limitations are largely patient-related rather than operator-related alone, underscoring that even technically well-performed examinations may be inadequate in certain populations. These findings highlight that, in a meaningful subset of at-risk individuals, ultrasound may be intrinsically suboptimal for surveillance and that such limitations should be explicitly documented in the imaging report to guide subsequent clinical decision-making. 

 The only large randomized controlled trial on HCC surveillance was conducted in China in 2004 and enrolled nearly 20,000 patients with chronic HBV infection. Participants were randomized to semiannual surveillance (ultrasound plus alpha-fetoprotein (AFP) test) or no surveillance. Despite suboptimal adherence (<60%) in the surveillance arm, significant survival benefits were observed: 66% at 1 year, 53% at 3 years, and 46% at 5 years, compared to 31, 7, and 0%, respectively, in the control group^142^. Given these striking results, similar studies in Western populations are now considered ethically infeasible. Ultrasound remains the most accessible, cost-effective, and widely used tool for routine monitoring in chronic liver disease^98^. Despite its advantages, the real-world effectiveness of ultrasound is often compromised by inconsistent surveillance intervals, poor-quality equipment, and limited operator expertise. These limitations contribute to the underdiagnosis of early-stage HCC in at-risk patients^27,107,117,128^. 

 Alternative imaging modalities, such as computed tomography (CT) scan or magnetic resonance imaging (MRI), have been proposed for HCC surveillance^70^. A Korean study demonstrated that MRI achieved substantially higher sensitivity than ultrasound (84.8 vs. 27.3%) and a significantly greater positive predictive value for detecting lesions smaller than 2 cm, while also yielding a markedly lower false-positive rate^70^. Similar findings were reported in a prospective French study, which likewise showed superior performance of MRI compared with ultrasound for early HCC detection^93^. However, cost, radiation exposure (in CT), and logistical barriers limit their widespread application. 

 Abbreviated MRI protocols have gained attention as a potential surveillance option in higher-risk populations — particularly patients with parenchymal heterogeneity due to obesity or advanced fibrosis, which can impair ultrasound accuracy^106,123^. However, robust evidence supporting their routine use is still lacking, and it is prudent to await the results of ongoing prospective clinical trials (NCT05828446; NCT05716620; NCT05657249; NCT05486572; NCT05095714; NCT04455932; NCT04288323), which are expected to provide more definitive data regarding diagnostic accuracy, clinical effectiveness, and cost-effectiveness before broad implementation in clinical practice can be recommended. 

### Recommendation 13

 Abdominal ultrasound is the first-line imaging modality for HCC surveillance. Evidence level: Moderate. Recommendation grade: Strong. 

### Should alpha-fetoprotein be used for hepatocellular carcinoma surveillance?

 AFP was historically the only available serological biomarker for HCC surveillance. However, when used alone, AFP has suboptimal sensitivity for early-stage tumors, as levels may remain within normal range in a significant proportion of patients^38^. Although its impact on overall survival remains uncertain, major guidelines currently endorse semiannual surveillance using abdominal ultrasound, with or without AFP, in high-risk populations^40,85,96,104^. 

 Recent evidence has explored the accuracy of this combined approach. A 2018 meta-analysis^128^ including 32 cohort studies (1990–2016) reported that while ultrasound alone had a sensitivity of 84% (95%CI 76–92%) for detecting HCC at any stage, it dropped to 47% (95%CI 3361%) for early-stage tumors. In comparative analyses, ultrasound combined with AFP showed higher sensitivity for any-stage HCC than ultrasound alone (RR 0.88; 95%CI 0.83–0.93). The advantage was even more pronounced for early-stage tumors, where sensitivity improved from 45% (95%CI 30–62%) with ultrasound alone to 63% (95%CI 48–75%) with AFP (p=0.002). However, this increase in sensitivity came at the cost of slightly lower specificity (RR 1.08; 95%CI 1.05–1.09), indicating a potential increase in false positives. 

 A second meta-analysis, published in 2021 by Colli et al.^24^, used a bivariate model to evaluate six studies that assessed ultrasound in combination with AFP using a 20 ng/mL cut point. The pooled sensitivity was 96% (95%CI 88–98%), and specificity was 85% (95%CI 73–93%). However, the authors highlighted that all the studies included were at high risk of bias, resulting in low-certainty evidence despite promising diagnostic performance. 

 Taken together, these findings suggest that while AFP should not be used as a standalone surveillance test, its addition to ultrasound modestly improves early-stage HCC detection and may be particularly helpful in high-risk patients. 

### Recommendation 14

 Serum alpha-fetoprotein should not be used as a standalone method for HCC surveillance. However, its combination with abdominal ultrasound may increase sensitivity for early tumor detection and can be considered, particularly in high-risk patients. Evidence level: Moderate. Recommendation grade: Weak. 

### What is the ideal interval for hepatocellular carcinoma surveillance?

 Two key studies help define the optimal interval. The first, conducted by an Italian group, analyzed 649 cirrhotic patients (Child-Pugh A and B) diagnosed with HCC between 1987 and 2006 during surveillance at 6- or 12-month intervals. Patients who underwent semiannual surveillance (78.6% of the cohort) were diagnosed with earlier-stage HCC, with smaller tumors (≤2 cm), and had significantly better outcomes, including a higher rate of curative treatment and improved overall survival (45 months vs. 30 months; p=0.001) compared to those screened annually^112^. The second study, a French multicenter trial, included 1,200 patients with cirrhosis who were randomized to 3- or 6-month ultrasound surveillance. The study found no survival benefit with the shorter interval^126^. Additionally, cost-effectiveness analyses have consistently supported the 6-month interval over both shorter and longer surveillance schedules^4^. 

### Recommendation 15

 HCC surveillance should be performed every 6 months. Evidence level: Moderate. Recommendation grade: Strong. 

### How should ultrasound reports be standardized for hepatocellular carcinoma surveillance?

**Figure 2:**
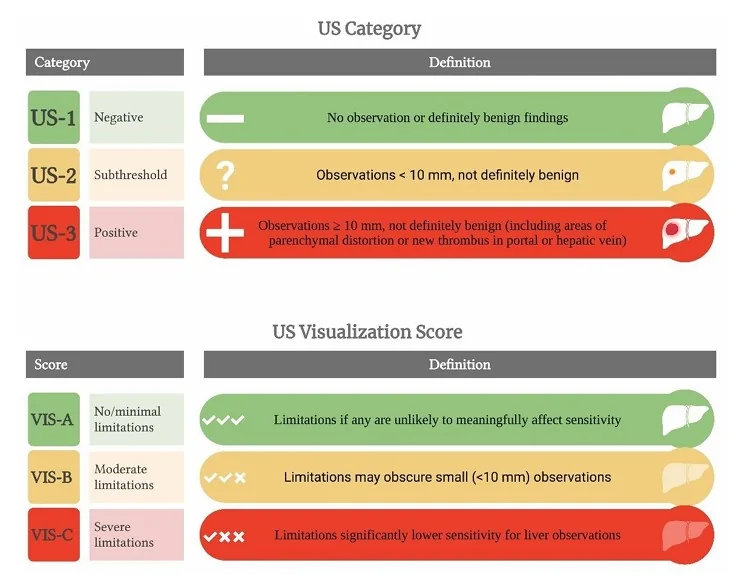
Summary of the LI-RADS^®^ Ultrasound Surveillance (Version 2024).

 Standardization of ultrasound reporting is essential to ensure consistent quality in HCC surveillance. The American College of Radiology formalized a dedicated ultrasound surveillance system for populations at risk of HCC in 2017 with the introduction of LI-RADS US LI-RADS^®^. A randomized prospective study conducted in Brazil^26^ evaluated the performance of conventional ultrasound versus a structured liver protocol based on US LI-RADS^®^. Among the patients assessed by trained physicians, the structured approach significantly improved detection: 10% (23 nodules/230 patients) vs. 1.3% (3 nodules/235 patients) with standard ultrasound (p<0.001). More recently, the American College of Radiology released the 2024 update of the LI-RADS ultrasound surveillance system^60^, summarized in Figure 2. 

 A systematic search for focal liver lesions is mandatory, with detailed characterization of each finding, including the size and morphology^35,38,85,108^. In addition, reports should explicitly document any acoustic limitations that may impair adequate visualization of the liver and compromise lesion detection^26,90,120,125^. 

 In addition to lesion detection, the report should include a Doppler assessment of hepatic vascular structures. Evaluation of the hepatic veins and portosplenic-mesenteric venous system is recommended to detect thrombosis, signs of portal hypertension (such as venous dilation or altered flow direction), splenic size and echotexture changes, and collateral circulation in the left gastric and perisplenic regions. The presence or absence of paraumbilical vein recanalization should also be documented^1,86^. 

### Recommendation 16

 Ultrasound reports used for HCC surveillance in highrisk patients should be structured and standardized as LIRADS US^®^, explicitly documenting any acoustic limitations that may impair liver visualization and lesion detection. The report must clearly state the presence or absence of focal hepatic lesions and describe nodules with indeterminate morphology, specifying whether they are smaller or larger than 1 cm. In high-risk patients, ultrasound alone should not be used to establish a definitive diagnosis of hepatic hemangioma, and additional imaging evaluation is required. Color and spectral Doppler assessment of hepatic and portal vascular structures should be routinely included to evaluate for portal hypertension and to identify possible tumor-related or hematologic thrombosis.Evidence level: Low. Recommendation grade: Strong. 

### What is the recommended approach for nodules smaller than 1 cm detected during hepatocellular carcinoma surveillance?

 The detection of subcentimeter nodules on ultrasound in cirrhotic patients remains a significant diagnostic challenge. While liver biopsy might seem a logical step, it has significant limitations in this context. In addition to cost and invasiveness, there is a low risk of complications, including bleeding and tumor seeding along the needle tract^9,89,115,135^. Moreover, due to the small size of these lesions, sampling errors are common, and histological interpretation may be inconclusive. Even for nodules under 2 cm, false-negative biopsy results may reach 30%^38^. 

 The 1 cm threshold was established because such lesions have a low probability of HCC^68^. These lesions tend to grow slowly, and available evidence does not indicate worse clinical outcomes for an 8 mm nodule compared with a 12 mm nodule^12^. Although nodules <1 cm may demonstrate arterial phase hyperenhancement on dynamic imaging, the positive predictive value and specificity for HCC at this size are relatively low. For this reason, LI-RADS is applied to lesions <1 cm, and these nodules may be categorized up to LR-4, but not LR-5, reflecting the limited diagnostic certainty in this size range. Dynamic imaging for nodules <1 cm may increase sensitivity but reduces specificity, potentially leading to false-positive diagnoses. Attempts to lower diagnostic thresholds in oncology to enable earlier treatment often lead to overdiagnosis without proven clinical benefit^105^. 

 Given the low probability of malignancy and the limitations of biopsy and imaging, short-interval ultrasound follow up is recommended. 

### Recommendation 17

 In patients with nodules smaller than 1 cm detected on ultrasound, follow-up should be performed at 3-4-month intervals for the first 2 years. If the nodule size remains stable after this period, follow-up can be resumed every 6 months. Evidence level: Low. Recommendation grade: Strong. 

### In which situations should abdominal magnetic resonance imaging or computed tomography be used for hepatocellular carcinoma surveillance?

 Although MRI and CT offer superior sensitivity and specificity compared to ultrasound, they are not routinely recommended for HCC surveillance in patients with cirrhosis. These tests have limited availability, higher cost, and potential adverse effects^109^. Additionally, although the risk of false positives is lower than with ultrasound, it remains a concern in population-based surveillance, and the overall cost-effectiveness is unfavorable for routine use^69^. 

 Nonetheless, MRI or CT may be considered in selected cases where ultrasound has significant technical limitations — such as in patients with obesity, nodular liver surface, excessive bowel gas, thoracic wall deformities, or in those awaiting liver transplantation, where accurate lesion detection is critical^36^. 

### Recommendation 18

 In patients with cirrhosis, MRI or CT may be considered as a surveillance modality for HCC when ultrasound has technical limitations that compromise its diagnostic performance. Evidence level: Low. Recommendation grade: Strong. 

## DISCUSSION

 This consensus represents a coordinated effort by multiple Brazilian medical societies to translate the best available scientific evidence into practical recommendations for the prevention and early detection of HCC. 

 Rather than serving as a prescriptive rulebook, a consensus document should be interpreted as a clinical framework designed to support informed decision-making127. Preventive and surveillance strategies must be individualized, considering each patient’s clinical profile, social circumstances, and values, as well as the realities of local healthcare delivery. Variations in resource availability, access to imaging modalities, regional expertise, and patient preferences may require adaptation of these recommendations to ensure that care remains safe, equitable, and centered on individual needs. 

 Although viral hepatitis still accounts for a significant portion of HCC cases, the widespread adoption of universal HBV vaccination and the increasing availability of highly effective DAA for HCV have started to change the epidemiologic landscape. Simultaneously, the rising prevalence of obesity, diabetes, and metabolic syndrome has made MASLD an increasingly important cause of HCC, especially in the Western world, including Brazil^43^. This epidemiologic shift suggests that much of the historical evidence — mostly based on cohorts dominated by viral hepatitis — may not fully represent the characteristics of future HCC populations. Therefore, prevention strategies are likely to evolve beyond solely controlling viral infections, with a greater focus on modifying metabolic risk factors. As the MASLD burden continues to grow, weightloss strategies, bariatric surgery, and modern pharmacologic treatments for obesity, including agents targeting the GLP-1 and GIP pathways, are expected to become key components of comprehensive HCC prevention^65,121^. 

 Despite important advances, substantial evidence gaps persist worldwide and are particularly pronounced in Brazil. Randomized clinical trials addressing HCC prevention remain scarce, and many recommendations necessarily rely on observational data that may be subject to residual confounding. Several clinically relevant questions remain unresolved, including the timing of antiviral therapy and the most appropriate risk-based surveillance strategies for patients with noncirrhotic MASLD. In addition, cost-effectiveness analyses of surveillance modalities tailored to the low- and middle-income countries (LMICs) are scarce, despite marked regional differences in infrastructure and resource availability. 

### Implementation challenges in low- and middle-income countries

 The translation of evidence-based recommendations into routine clinical practice is particularly challenging in LMICs, where healthcare systems often face structural constraints, uneven resource distribution, and limited access to specialized care. In such settings, disparities in diagnostic capacity, availability of trained personnel, and referral networks may hinder timely surveillance and early detection of HCC. Even when national guidelines exist, implementation may be inconsistent across regions due to differences in infrastructure, funding mechanisms, and healthcare organizations. 

 Equity of access to healthcare is a critical prerequisite for the meaningful implementation of any evidence-based recommendation. Well-formulated guidelines have limited practical value if clinicians lack access to the diagnostic tools, treatments, or referral pathways required to apply them. Technical limitations of imaging-based surveillance further compound this challenge. Ultrasound performance is influenced by operator expertise, liver morphology, obesity, and equipment quality, all of which may compromise early tumor detection in routine practice. These limitations underscore the need for standardized reporting and targeted training initiatives. 

 Finally, it is essential to acknowledge that the evidence underpinning most current HCC prevention and surveillance strategies is derived mainly from studies conducted in high-income countries. The relative paucity of data generated in low- and middle-income settings limits the ability to fully account for regional differences in demographics, genetics, environmental exposures, socioeconomic conditions, and healthcare system organization, which may not be adequately represented in existing cohorts. Expanding research capacity and generating high-quality data from diverse global settings are therefore essential to improve the external validity and global applicability of preventive and surveillance strategies, and to ensure that future consensus recommendations are responsive to the heterogeneity of HCC care across different health systems and populations worldwide. 

## CONCLUSIONS

 This multidisciplinary Brazilian consensus provides recommendations for the prevention and surveillance of HCC, combining current evidence and clinical expertise. By reinforcing prevention and early detection strategies, this guidance aims to reduce the burden of HCC and support improved clinical outcomes across diverse healthcare settings. 

## Data Availability

The datasets generated and/or analyzed during the current study are available from the corresponding author upon reasonable request.
